# Why Serological Responses during Cystitis are Limited

**DOI:** 10.3390/pathogens5010019

**Published:** 2016-02-14

**Authors:** Hae Woong Choi, Soman N. Abraham

**Affiliations:** 1 Departments of Pathology, Duke University Medical Center, Durham, NC 27710, USA; 2 Immunology Duke University Medical Center, Durham, NC 27710, USA; 3 Molecular Genetics & Microbiology Duke University Medical Center, Durham, NC 27710, USA; 4 Program in Emerging Infectious Diseases Duke-National University of Singapore, Singapore 169857, Singapore

**Keywords:** recurrent UTI, cystitis, mast cells, defects in memory response, serology

## Abstract

The high frequency of urinary tract infections (UTIs), some of which appear to be endogenous relapses rather than reinfections by new isolates, point to defects in the host’s memory immune response. It has been known for many decades that, whereas kidney infections evoked an antibody response to the infecting bacteria, infections limited to the bladder failed to do so. We have identified the existence of a broadly immunosuppressive transcriptional program associated with the bladder, but not the kidneys, during infection of the urinary tract that is dependent on bladder mast cells. This involves the localized secretion of IL-10 and results in the suppression of humoral immune responses in the bladder. Mast cell-mediated immune suppression could suggest a role for these cells in critically balancing the needs to clear infections with the imperative to prevent harmful immune reactions in the host.

## 1. Introduction

UTIs are the second most common bacterial infection in the body, accounting for about 8.1 million doctor visits annually, the majority of which occur in women where a significant number appear to be recurrent [1]. Over 80 percent of infections are caused by uropathogenic *E. coli* (UPEC). Although virulence factors such as adhesive fimbriae have contributed significantly to UPEC pathogenesis [2,3,4,5,6] predisposing host factors also contribute to UTIs, particularly in individuals prone to recurrent episodes.

A prominent and long recognized host anomaly relates to the apparent inability of patients experiencing cystitis to evoke an antibody response to the infecting bacteria, unlike their counterparts suffering pyelonephritis [7,8,9,10]. This is especially surprising as the early innate immune responses to bladder infections in these patients are invariably rapid and vigorous [11,12,13]. The distinct inability of the bladder to evoke a memory response was even the basis of a diagnostic test to distinguish cystitis patients from those experiencing pyelonephritis. The test involved examining for antibody-coated bacteria in urine specimens of UTI patients [14,15]. If bacteria found in urine were coated with antibody it was indicative of pyelonephritis whereas if the bacteria were not antibody coated, it was assumed that the patient suffered cystitis [14,15]. In spite of its enormous clinical relevance, few studies have been undertaken to investigate why antibody responses during cystitis are limited.

## 2. Recapitulation of Clinical Findings in a Mouse Model

Recently, we sought to investigate the underlying basis for this striking anomaly in the immune defenses of the urinary tract [16]. Initially, we sought to recapitulate the inability of the bladder to evoke an antibody response following cystitis. For this experiment, it was important to ascertain that we could cause infections exclusively in the bladder of mice in addition to infections involving both the bladder and kidneys. We found that by administering 30 μL of a suspension containing 10^8^ cells of *E. coli* strain CI5 directly into the bladder via a catheter, we could evoke a bladder infection without any bacteria ascending up via the ureter into the kidney [16]. It was important to instill the fluid slowly into the bladder to prevent reflux into the kidneys. To cause kidney infections we instilled a comparable dose of bacteria in 50 μL of fluid. We administered this fluid briskly ensuring that bacteria reached the kidneys at the time of instillation [16]. In this manner, groups of female mice were given exclusively bladder infections or bladder and kidney infections. On days 7, 14 and 21 post infection (p.i.) we collected serum from the infected mice and examined for the presence of UPEC-specific antibody by ELISA. We found that in contrast to animals given exclusively bladder infections, mice with pyelonephritis evoked a significant bacteria-specific IgG antibody response by day 7 which subsided by day 21 indicating a rapid but short lived serological response [16]. Thus, this mouse model revealed that there was a site-specific defect in the development of primary adaptive immunity in bacterial infections limited to the bladder similar to the human clinical picture.

**Figure 1:**
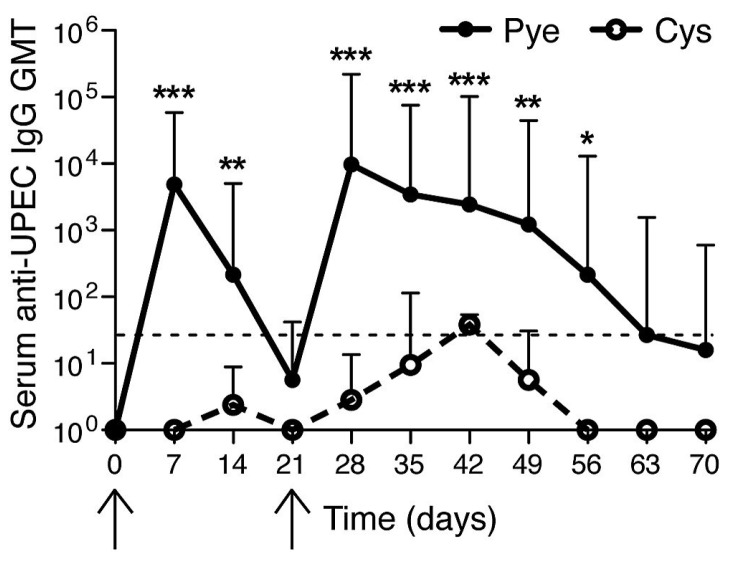
Failure of humoral responses in bladder during uropathogenic *E. coli* (UPEC) infection. Serum geometric mean titers (GMT) of UPEC antibodies were measured. (First arrow) 1 × 10^8^ CFU UPEC was transurethrally given to mice bladder for inducing cystitis-only or pyelonephritis. (Second arrow) both groups were infected through cystitis-only. * *p* < 0.05; ** *p* < 0.01; *** *p* < 0.001. *n* = 4–7. Dotted line: detection threshold. This figure is a reproduction from [16].

We next sought to examine if indeed the mouse bladder was defective in evoking a memory response by examining whether or not previously infected bladders would evoke a serologic response after a second infection. For these studies, either a bladder restricted infection (cystitis) or combined bladder and kidney infection (pyelonephritis) were induced in mice on day 0 (Figure 1, first arrow). Serum antibodies against *E. coli* were measured p.i. using ELISA. We observed high anti-*E. coli* titers in mice with pyelonephritis and a lack of substantial *E. coli* antibody titers in mice having cystitis. On day 21 (Figure 1, second arrow) both groups of mice (cystitis alone *vs.* pyelonephritis) were reinfected but employing the cystitis alone model, with the same *E. coli* strain as the primary challenge. As expected, upon a second challenge, mice with prior pyelonephritis showed a strong secondary antibody response to UPEC (Figure 1). Although higher titers compared to the primary response (a hallmark of memory recall response) were not observed at the time points assayed, this memory response was characterized by a prolonged duration of detectable antibody responses compared to the first infection (Figure 1). However, mice that had a primary infection restricted to the bladder showed no significant (*p* > 0.05) memory recall response to cystitis rechallenge (Figure 1) pointing to tempering of the adaptive immune response in the bladder that does not follow the normal pattern for a secondary humoral response. These observations suggest that if adaptive responses have already been established, the bladder is immunologically competent to respond, whereas a primary infection of the bladder alone has limited ability to initiate immunological memory.

## 3. The Bladder unlike the Kidneys Evokes a Muted Antibody Response during Infection

**Figure 2:**
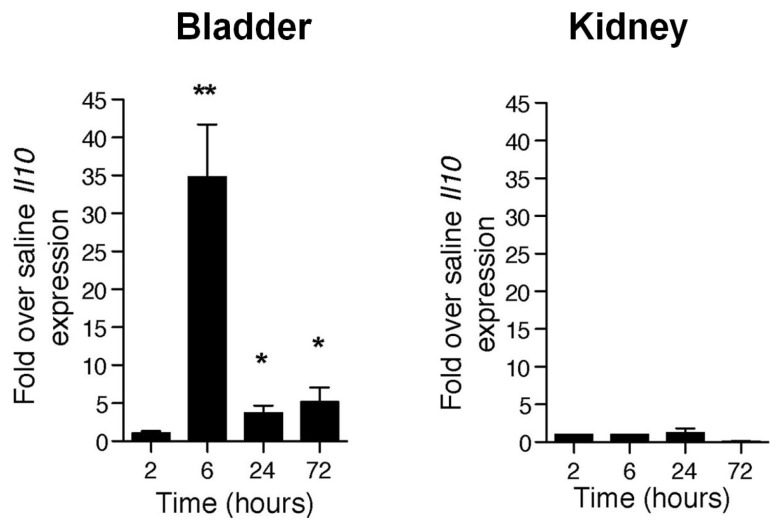
UPEC infection specifically enhanced *Il10* transcription as an anti-inflammatory response in the bladder. *Il10* transcription levels (real-time PCR) in the bladder and kidney measured at various times p.i. * *p* < 0.05; ** *p* < 0.01. This figure is a reproduction from [16].

To elucidate why the bladder’s response to UPEC infection is so different from that of the kidneys, we studied the cytokine profile of both organs following UPEC infection. As expected we observed a strong proinflammatory response early in the infection with drastic upregulation of mRNA for inflammatory mediators such as IL-1, CXCL1 and TNF in the bladders of mice experiencing only cystitis or cystitis and pyelonephritis [16]. However, 6 h p.i. there suddenly appeared an upregulation of message for a major inhibitor of proinflammatory responses, IL-10, in the bladder, which was distinctly absent in the kidneys (Figure 2). This observation reveals that, subsequent to the strong proinflammatory innate responses, the bladder specifically responds to UPEC infection with a second muted phase of response involving the induction of a prominent anti-inflammatory regulator, IL-10.

To discern the functional consequence of IL-10 production during bladder infection, we infected IL-10 deficient mice (IL-10^−/−^) with *E. coli*, using our model of cystitis and determined that the serological response to cystitis infected mice was now comparable to that seen in the mice experiencing pyelonephritis [16]. Since IL-10^−/−^ mice experience a class switch defect [17,18], we assayed for IgM antibodies, rather than IgG. This observation implicates IL-10 in diminishing the serological response to *E. coli* following bladder infection.

**Figure 3:**
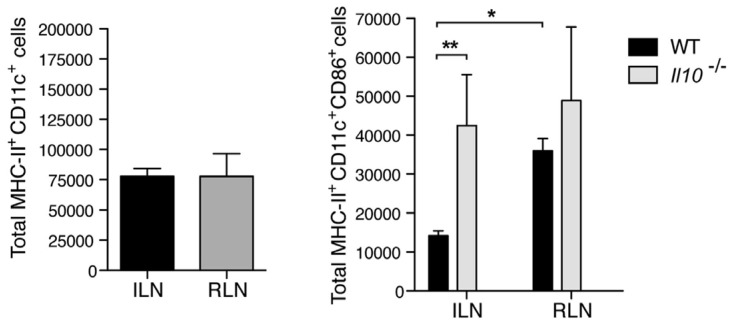
IL10 suppresses the activation of dendritic cells during cystitis. (**Left**) Upon cystitis or pyelonephritis, MHC-II^+^CD11c^+^ population of DCs were measured by flow cytometry after 24 h p.i.; (**Right**) Increased population of MHC-II^+^CD11c^+^CD86^+^ DCs of WT in RLN or *Il10^−/−^* in ILN and RLN. ILN: iliac lymph node; RLN: renal lymph node; * *p* < 0.05; ** *p* < 0.01; *n* = 3–7. This figure is a reproduction from [16].

To investigate the mechanism underlying IL-10 mediated suppression of antibody responses in the bladder, we examined the early cellular events associated with development of a serological response. Since dendritic cells at the site of infection are key mediators of the antibody response, we examined the migration and activation state of dendritic cells into the lymph nodes draining the bladder (iliac) and kidneys (renal) in wild type and IL-10^−/−^ mice. We found that, although there was no appreciable difference in the trafficking of dendritic cells from the bladder to the iliac lymph nodes, the proportion of activated CD86^+^ dendritic cells were significantly lower in the lymph nodes of IL-10^−/−^ mice compared to wild type. This was not the case with regard to dendritic cells flowing into the renal lymph nodes (Figure 3). Therefore, increase of IL-10 in the bladder upon infection influences the activation of lymph node draining dendritic cells and may explain the uniquely dampened antibody responses to bladder infection.

## 4. Mast Cells Mediate the IL-10 Dependent Suppression of Antibody Responses in the Bladder

**Figure 4:**
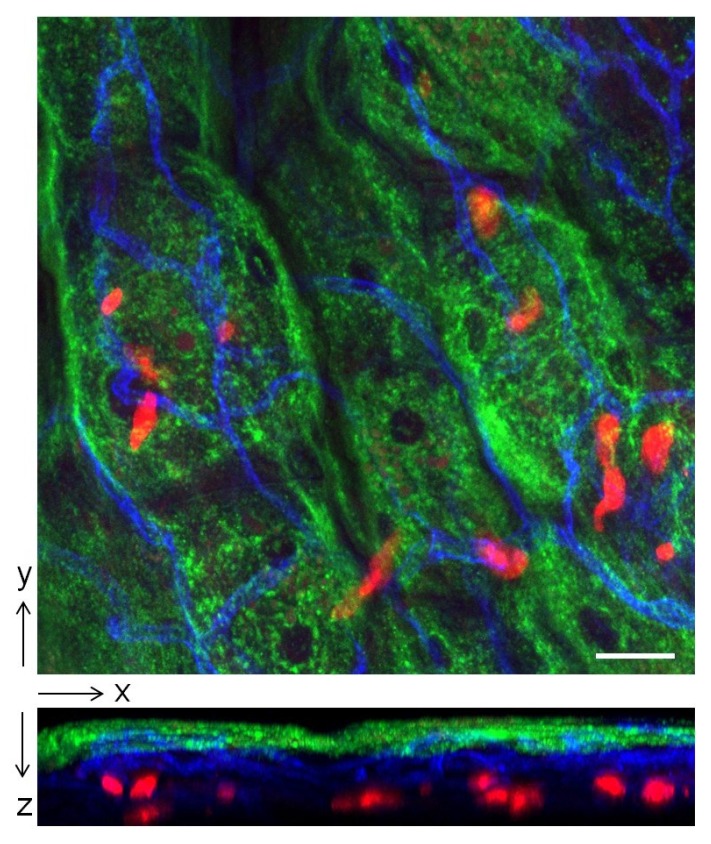
Localization of mast cells in the epithelium of the bladder. Bladder of Mcpt5-Cre *tdTomato*^fl/fl^ mouse was whole-mounted and stained. Green: superficial epithelial cells (wheat germ agglutinin); Blue: blood vessels (anti CD31 antibody); Red: mast cells (tdTomato); Scale bar: 30 µm. Upper figure is a top view image showing spatial distribution of mast cells relative to superficial epithelial cells and blood vessels. Lower figure is a side view image revealing the close proximity of mast cells to the superficial epithelium and blood vessels, where immune cells are recruited from.

Having established the role of IL-10 in immune suppression in the bladder we were interested in identifying the cellular source of IL-10. A major immune cell capable of producing IL-10 during inflammation is the mast cell. In addition to its well-known inflammatory actions these cells play largely overlooked roles in maintaining tissue homeostasis [19,20]. For example they have been reported to limit inflammation during contact hypersensitivity through secretion of IL-10 [19]. Mast cells are immune surveillance cells that are especially abundant underneath the epithelium of the bladder (Figure 4). Because of their strategic location and their capacity to promptly release many prestored inflammatory mediators, they are in a position to critically modulate immune responses in the bladder. By comparing neutrophil responses in wild type and mast cell deficient (*W/W^v^*) mice it was deduced that these cells were important in promoting the early neutrophil recruitment to sites of bladder infection [21]. To see if mast cells were the cellular source of IL-10 at the 6 h point after infection, we compared IL-10 expression in the bladders and kidneys of wild type and mast cell deficient (Wsh) mice following cystitis and pyelonephritis. We found that whereas a sharp and significant increase in IL-10 expression was seen in the bladder of wild type mice, no such spike in IL-10 expression was seen in Wsh mice. ELISA for IL-10 in the urine of wild type mice and Wsh mice with bladder infections confirmed this finding [16]. As expected, no spike in IL-10 expression was seen in the kidneys of both groups of mice after infection [16]. Cumulatively, these observations point to mast cells as being the critical cellular source of IL-10 at the 6 h p.i. time point.

To verify that the IL-10 production in the bladder during infection is indeed mast cell dependent, we reconstituted Wsh mice with bone marrow derived mast cells from wild type and from IL-10^−/−^ mice. We found mice reconstituted with wild type but not IL-10^−/−^ derived mast cells evoked IL-10 responses following bladder infection. Since some findings employing the Kit dependent models of mast cell deficiency (including Wsh mice), have recently been questioned [22,23], we sought to examine the contribution of mast cells to bladder IL-10 response employing a Kit independent model of mast cell deficiency. We utilized a Cre-LoxP recombination model with the Cre gene under the regulation of the mast cell protease-5 (Mcpt5) promoter and *Il10* gene flanked with loxP sites [23].

**Figure 5:**
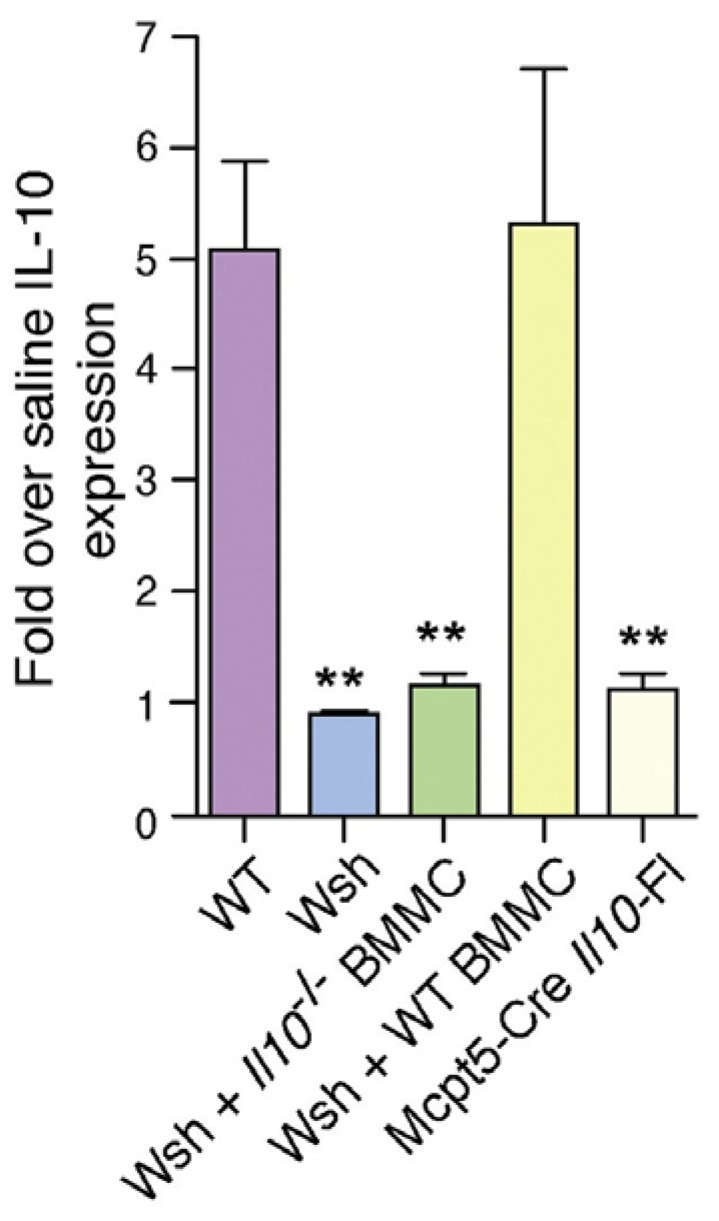
Bladder mast cells express IL10 during UPEC infection. Individual group of mice were infected with UPEC, and at 24 h p.i. *Il10* expression (real time PCR) was measured. ** *p* < 0.01. This figure is a reproduction from [16].

**Figure 6:**
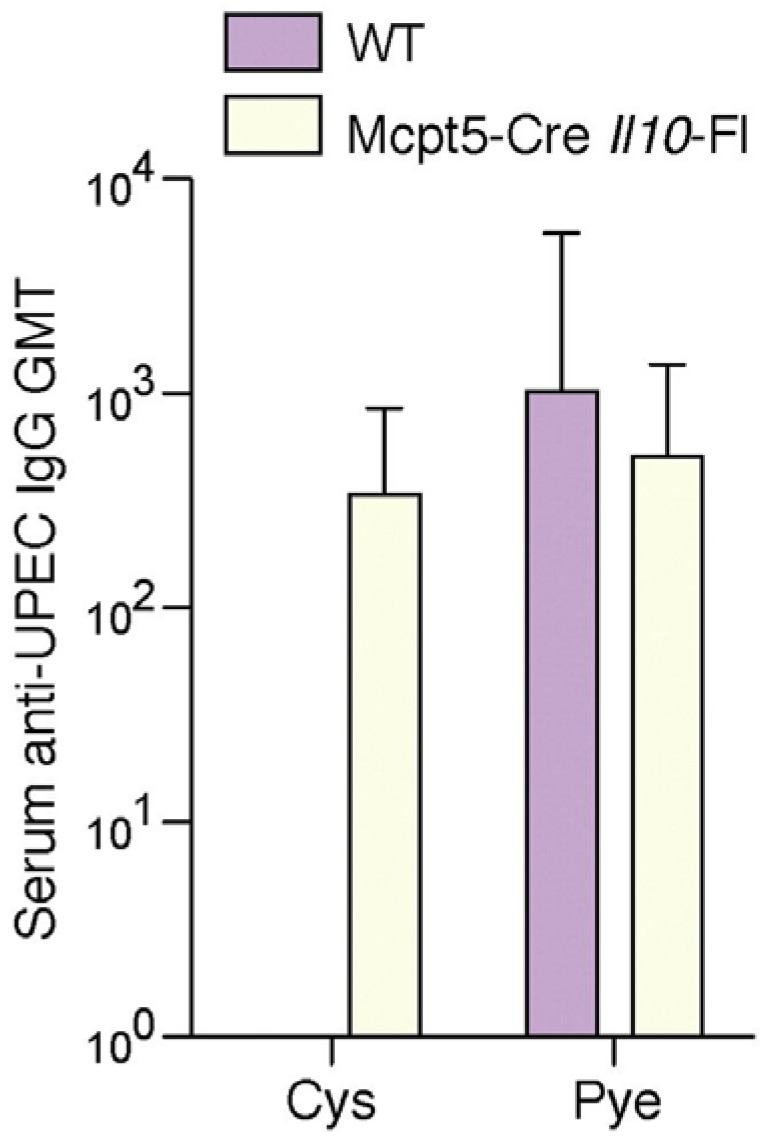
UPEC-specific IgG production is no longer suppressed in mice whose mast cells fail to secrete IL-10. Serum UPEC-specific IgG was detected in Mcpt5-Cre *Il10^fl/fl^* mice 7days p.i. but not in wild type mice. *n* = 5; Error bars represent the 95% confidence level. This figure is a reproduction from [16].

In these mice, only mast cells were deficient in IL-10 (Mcpt5-Cre*il10*^fl/fl^) [16]. In Mcpt5-Cre*il10*^fl/fl^ mice we observed diminished expression of IL-10 at the 6 h time point following infection which was consistent with findings using Kit mast cell deficiency model (Figure 5). Additionally, we examined the sera of these Mcpt5-Cre *Il10*^fl/fl^ mice following bladder infection, we observed a distinct *E. coli* specific antibody response which was in contrast to wild type mice (Figure 6). As expected no difference was detected in the antibody response to bacteria following kidney infection in wild type and Mcpt5-Cre *I10*^fl/fl^ mice (Figure 6). Cumulatively, these studies indicate the defective serological response to bladder infection is attributable to immune suppressive actions of mast cell mediated IL-10.

## 5. Why do Bladder Mast Cells Secrete IL-10 to Suppress Immune Responses and Specifically at 6 h p.i.?

At this time there is no data to answer these questions but we speculate that a major reason for why mast cell’s switch from a proinflammatory state to an anti-inflammatory one is to protect the integrity of the epithelial barrier of the bladder and to prevent the development of memory responses to the host antigens found in the urine. The bladder has a unique storage function, which is retention of urine for significant periods before it is voided. Urine contains many noxious and toxic compounds as well as potentially immunogenic breakdown products of host proteins that could evoke harmful immune responses. These detrimental effects are normally prevented by an impermeable and intact superficial epithelium. However, a powerful defensive reaction to bacterial infection in the bladder is spontaneous exfoliation of the superficial epithelium which appears to be a highly effective mechanism to rapidly reduce bacterial load. Indeed, the presence of large swathes of shed epithelial cells with adherent bacteria in the urine is often diagnostic of UTI. Even though exfoliation of epithelial cells rapidly reduces the bacterial load in the bladder it also leaves the underlying tissue exposed to the highly toxic compounds in urine. This harmful situation could be averted or its impact reduced if the bladder epithelium were able to promptly regenerate. Indeed, the bladder epithelium is programmed to rapidly regenerate its superficial layer immediately following exfoliation. Upon shedding of the superficial layer, stem cell-like cells in the basal layer of the urothelium secrete factors triggering rapid proliferation of uroepithelial and stromal cells resulting in the reformation of a tight impregnable barrier of superficial epithelia cells [24]. However these repair mechanisms will be limited if inflammatory reactions are still at its heightened state. Presumably the mast cell IL-10 response is directed at tempering the inflammatory milieu in the bladder epithelium to allow repair processes to occur. It is noteworthy that the spike in mast cell IL-10 production in the bladder is detected only after 6h p.i. which is significant as that corresponds with when shedding of superficial bladder epithelial cells begins [25]. It would appear that the curtailing of adaptive immune responses to the infecting bacteria in the bladder is an unintended consequence of the tempering action of IL-10. On the other hand, curtailing of immune responses is beneficial as it will prevent the exposed bladder tissue from evoking potentially harmful immune responses to self-antigens found in urine. At this time the notion that the mast cell IL-10 response is attributable to the breakdown of the epithelial barrier following infection remains speculative. One approach to investigating this question will be examining the immune responses to bladder infections in mice defective in their ability to shed superficial epithelium of the bladder.

## 6. Conclusions

Our findings finally provide an explanation for the clinical observed as to why patients with bladder infections fail to evoke an antibody response to the infecting bacterial strain, a significant contributing factor for the high frequency of recurrence of bladder infections. Employing mouse models of infection, we have linked the abrogation of the adaptive immune response following bladder infection to the abrupt production of anti-inflammatory cytokine IL-10 by local mast cells. The inability of the bladder to mount an antibody response, however, does not preclude the employment of vaccination strategies to combat UTIs. Most vaccines directed at virulence factors of UPEC are typically administered at sites other than the bladder therefore, the constraints described here should not apply. Indeed, vaccines comprising of UPEC virulence factors such as fimbrial proteins and siderophore receptors have been shown to be highly efficacious against UTIs in mouse models [26,27]. The inability of the bladder to mount a meaningful adaptive response appears the result of the bladder choosing the lesser of two evils: prematurely aborting memory immune responses over leaving the bladder unprotected from harmful toxic materials in urine. The critical “decider” in this scenario appears to be mast cells which are functionally versatile and strategically positioned in significant numbers under the basal membrane of the bladder epithelium to both sense bacterial challenge and direct the most appropriate cellular host responses an any given time during the infection.
